# Prognostic value of forward flow indices in primary mitral regurgitation due to mitral valve prolapse

**DOI:** 10.3389/fcvm.2023.1076708

**Published:** 2023-02-23

**Authors:** Elisabeth Petolat, Alexis Theron, Noemie Resseguier, Cyprien Fabre, Giulia Norscini, Rita Badaoui, Gilbert Habib, Frederic Collart, Stéphane Zaffran, Alizée Porto, Jean-François Avierinos

**Affiliations:** ^1^Department of Cardiology, La Timone Hospital, Marseille, France; ^2^Department of Cardiac Surgery, La Timone Hospital, Marseille, France; ^3^EA 3279, Faculté de Médecine, Marseille, France; ^4^U1251 INSERM, Marseille Medical Genetics, Aix-Marseille University, Marseille, France

**Keywords:** mitral regurgitation, mitral valve prolapse, left ventricular dysfunction, stroke volume, mitral repair surgery

## Abstract

**Background:**

Degenerative mitral regurgitation (DMR) due to mitral valve prolapse (MVP) is a common valve disease associated with significant morbidity and mortality. Timing for surgery is debated for asymptomatic patients without Class I indication, prompting the search for novel parameters of early left ventricular (LV) systolic dysfunction.

**Aims:**

To evaluate the prognostic impact of preoperative forward flow indices on the occurrence of post-operative LV systolic dysfunction.

**Methods:**

We retrospectively included all consecutive patients with severe DMR due to MVP who underwent mitral valve repair between 2014 and 2019. LVOT_TVI_, forward stroke volume index, and forward LVEF were assessed as potential risk factors for LVEF <50% at 6 months post-operatively.

**Results:**

A total of 198 patients were included: 154 patients (78%) were asymptomatic, and 46 patients (23%) had hypertension. The mean preoperative LVEF was 69 ± 9%. 35 patients (18%) had LVEF ≤ 60%, and 61 patients (31%) had LVESD ≥40 mm. The mean post-operative LVEF was 59 ± 9%, and 21 patients (11%) had post-operative LVEF<50%. Based on multivariable analysis, LVOT_TVI_ was the strongest independent predictor of post-operative LV dysfunction after adjustment for age, sex, symptoms, LVEF, LV end systolic diameter, atrial fibrillation and left atrial volume index (0.75 [0.62–0.91], *p* < 0.01). The best sensitivity (81%) and specificity (63%) was obtained with LVOTTVI ≤15 cm based on ROC curve analysis.

**Conclusion:**

LVOT_TVI_ represents an independent marker of myocardial performance impairment in the presence of severe DMR. LVOT_TVI_ could be an earlier marker than traditional echo parameters and aids in the optimization of the timing of surgery.

## Introduction

Mitral valve prolapse (MVP) is a common valvular heart disease with a prevalence that approaches that of bicuspid aortic valve and is estimated to occur in between 0.6 and 2.4% of the population (1, 2). MVP has been the leading cause for severe primary mitral regurgitation (MR) requiring surgery in western countries for five decades (3). Mitral valve repair can be achieved in most patients, improving symptoms and restoring life expectancy when surgery is performed before left ventricular (LV) systolic dysfunction, which remains the ultimate complication of organic MR (4), the first cause of post-operative mortality (5) and the hinge point of the highly debated optimal timing for surgical correction. Severe degenerative MR (DMR) management is a controversial topic between defenders of a traditional symptom waiting approach (6) and supporters of earlier surgical strategies (7, 8). The latter note the challenge of early detection of LV impairment in the presence of severe MR-related altered loading conditions (9, 10), the high incidence of unexpected post-operative LV dysfunction despite normal preoperative LV parameters and observational data reporting restoration of outcome after early repair (11–13). In this context, both US and European guidelines rely on clinical and echo triggers to determine the timing for surgery (14, 15), noting that the post-operative outcome of symptomatic patients (16) and of those with preoperative decreased LV ejection fraction (EF) (10) or increased LV end-systolic diameters (LVESD) (9) might not be optimal. Therefore, the aforementioned guidelines open the door for earlier surgical strategies as class IIa indications in patients with no symptoms and normal LV echocardiographic parameters (14, 15) provided that repair is feasible in a high volume Heart Valve Center. However, European guidelines mention left atrial (LA) dilation as an additional condition (15), underlining the current ambiguity surrounding primary MR management. This controversy has prompted cardiologists to search for new indicators of earlier evidence of LV systolic dysfunction in patients with severe DMR to refine surgical timing in asymptomatic patients with normal classic LV echographic markers (17–20). Among those, we tested the hypothesis that in the presence of severe DMR, alterations in forward LV ejection markers, specifically LV outflow tract velocity-time integral (LVOT_TVI_) and stroke volume index (SVi), could represent simple and reproducible indicators of early LV systolic dysfunction, helping risk stratification and decision-making.

## Methods

### Study population and design

We conducted an observational retrospective single-center study that included all consecutive patients with severe primary degenerative MR (DMR) due to MVP who underwent MV repair between 2014 and 2019, whose forward SVi was available and whose post-operative follow-up (FU) was ≥6 months. Exclusion criteria were outside echocardiographic diagnosis without institutional full evaluation, secondary MR, mitral stenosis, significant aortic valve disease, previous valvular surgery, congenital heart disease and patients with incomplete or unavailable clinical or echo data. Coronary artery disease was not considered an exclusion criterion if it did not generate secondary MR. Patients who denied authorization for anonymous publication of their clinical data for research purposes were also excluded. The study was conducted in accordance with institutional guidelines, national legal requirements and the revised Declaration of Helsinki. All included patients provided written consent for research and publication of their study data (IRB approval number 2019-48).

### Clinical and echocardiographic data

Data from clinical examination, 12-lead ECG and transthoracic echocardiography (TTE) performed in our institution by experienced cardiologists within 3 months prior to surgery and at 6 months FU were available in all patients. Transthoracic echocardiograms were performed within routine clinical practice using standard methods (21, 22). LV and LA diameters and volumes were recorded in the long axis parasternal and apical views, and the left ventricular ejection fraction (LVEF) was estimated visually using the Simpson biplane method. The diagnosis of MVP was made as recommended (1), and the diagnosis of flail leaflet was based on failure of leaflet coaptation with rapid systolic movement of the flail segment into the LA (23, 24). MR severity was assessed following an integrative approach as recommended (22). Original data were used that were unaltered from the original prospective echocardiographic data collection by means of electronic transfer. The LV outflow tract (LVOT) diameter was measured in the parasternal long axis view, and LVOT_TVI_ was recorded as recommended (22) by pulse wave Doppler in the apical 5-chamber view. Three cardiac cycles at least in sinus rhythm and 10 in atrial fibrillation were averaged. Stroke volume (SV) was calculated as the product of LVOT area by LVOT_TVI_ and was indexed to body surface area (BSA) and referred to as SVi. A threshold of <35 ml/m^2^ was considered as *a priori* abnormal by reference to aortic stenosis (25). Forward LVEF was calculated as the ratio of LVOT stroke volume to LV end-diastolic volume (LVEDV), and a value <50% was considered abnormal (26).

### Statistical analysis

The endpoint was the occurrence of post-operative LV systolic dysfunction defined by an LVEF <50% at ≥6 months after MV repair (27). Quantitative variables were described as the means ± standard deviations, and qualitative variables were described as numbers and percentages. Non-normally distributed data were reported as median and interquartile ranges. For univariate analyses, comparisons between groups according to the primary endpoint were made using the Chi^2^ test if valid (Fisher's exact test otherwise) for the qualitative variables and using Student's *t*-test if valid (Mann–Whitney test otherwise) for the quantitative variables. For echocardiographic parameters, a univariate logistic regression model was used to estimate crude odds ratios with their 95% CIs quantifying the excess risk of post-operative LV dysfunction. Multivariate analyses were then performed to assess the independent effect of echocardiographic parameters while taking into account potential prognostic factors selected beforehand according to the literature data and to the results of univariate analysis. Adjusted odds ratios were estimated with their 95% CIs. Firth's correction was applied by performing Firth's penalized-likelihood logistic regression to take into account the small number of post-operative LV dysfunction.

Receiver operating characteristic (ROC) curves were established to determine the optimal cutoff value of echocardiographic parameters to predict post-operative LV dysfunction. Echocardiographic parameters were then dichotomized according to the identified cutoff values. Univariate and multivariate Firth's penalized-likelihood logistic regression models were built to estimate crude and adjusted odds ratios with their 95% CIs related to these dichotomized parameters for predicting post-operative LV dysfunction.

All tests were two-sided. All *p*-values<0.05 were considered significant. We used R version 4.0.5 for all statistical analyses.

## Results

### Preoperative data

One hundred ninety-eight patients were included between 2014 and 2019. The mean age was 64 ± 13 years. The majority of patients were males (74%), and 45 (23%) had a history of atrial fibrillation (AF), which was permanent in 22 (11%). The majority of patients were in NYHA class I-II (78%) at the time of surgery. The mean BNP level was 126 ± 133 pg/ml, and the Euroscore was low (Table 1). All patients had severe DMR as attested by quantitative parameters and the high prevalence of flail leaflets (mean effective regurgitant orifice (ERO) = 50 ± 15 mm^2^). The middle portion of the posterior leaflet (P2) was the most frequently involved segment either isolated or in combination with other locations. The mean preoperative LVEF was normal, but 35 (18%) patients had a preoperative LVEF ≤60%, and 2 (1%) had an LVEF<50%. The mean LVESD was 35 ± 7 mm, and 61 (31%) patients had LVESD ≥40 mm. The mean LA volume index (LAVI) was 72 ± 24 ml/m^2^, and 131 (66%) patients had an LAVI ≥60 ml/m^2^. The mean systolic pulmonary artery pressure (sPAP) was 40 ± 15 mmHg, and 36 (18%) patients had sPAP>50 mmHg. The mean LVOT_TVI_ was 16 ± 3 cm. The forward SVi was 37 ± 8 ml/m^2^, and 88 (44%) patients had a forward SVi <35 ml/m^2^. The mean forward LVEF was 38 ± 13%, and 167 (84%) patients had forward LVEF<50% (Table 2).

**Table 1:** Preoperative clinical characteristics of 198 patients subject to mitral valve repair for severe DMR.

	***n* = 198**
Age (year)	64 ± 13
Female (%)	51 (26)
Diabetes mellitus (%)	3 (1)
Hypertension (%)	46 (23)
Atrial fibrillation (%)	45 (23)
Chronic lung disease (%)	13 (6)
Chronic renal insufficiency (%)	2 (1)
**NYHA functional class (%)**	
I (%)	37 (18)
II (%)	117 (59)
III (%)	40 (20)
IV (%)	4 (2)
**Euroscore II (%)**	1.4 ± 1.1
BNP (pg/ml)	126 ± 133

**Table 2:** Pre-operative echocardiographic characteristics of 198 patients subject to mitral valve repair for severe DMR.

	***n* = 198**
MR ERO (mm)^2^	50 ± 15
MR RVol (mL)	97 ± 41
LVEDD (mm)	58 ± 7
LVESD (mm)	35 ± 7
Indexed LVEDD (mm/m^2^)	32 ± 4
Indexed LVESD (mm/m^2^)	19 ± 4
LVEDV (ml)	190 ± 53
LVESV (ml)	59 ± 24
Indexed LVEDV (ml/m^2^)	103 ± 25
Indexed LVESV (ml/m^2^)	32 ± 12
LVEF (%)	69 ± 9
LVOT_**TVI**_ (cm)	16 ± 3
Forward SV (ml)	68 ± 16
Forward SVi (ml/m^2^)	37 ± 8
Forward LVEF (%)	38 ± 13
Indexed LA volume (ml/m^2^)	72 ± 24
sPAP (mmHg)	40 ± 15
Mitral valve prolapse	
**Posterior leaflet**	
P1 (%)	29 (14)
P2 (%)	173 (87)
P3 (%)	42 (21)
**Anterior leaflet**	
A1 (%)	9 (4)
A2 (%)	31 (15)
A3 (%)	18 (9)
Flail leaflet (%)	143 (72)

### Surgical management

Surgery was indicated by symptoms or LVEF ≤60% or LVESD ≥40 mm (class I indications) in 102 (51.5%) patients and by AF or sPAP ≥50 mmHg while asymptomatic with no overt LV dysfunction (Class IIa indications) in 23 (11.5%). In addition, 73 (37%) patients had “early” class IIa indications (asymptomatic and sinus rhythm and sPAP <50 mmHg and LVEF >60% and LVESD < 40 mm).

All patients underwent MV repair, including 150 patients (76%) with neo-chordae and 48 patients (24%) with leaflet resection. An annuloplasty ring was implanted in all patients. The mean duration of cross-clamping time was 55 ± 18 min. Operative trans-TEE showed no residual MR in 99 (50%) patients, mild MR in 95 (48%) patients and moderate MR in 4 (2%) patients. The LVEF was 55 ± 9% at discharge. No operative deaths or post-operative strokes were noted.

### Incidence and determinants of post-operative LV dysfunction

TTE performed at 6 months after FU in all 198 patients showed a mean LVEF of 59 ± 9% with 21 (11%) patients displaying post-operative LV dysfunction. The vast majority of patients had no or trivial MR, 21 (11%) had mild MR, and none had moderate or severe MR.

**Figure 1:**
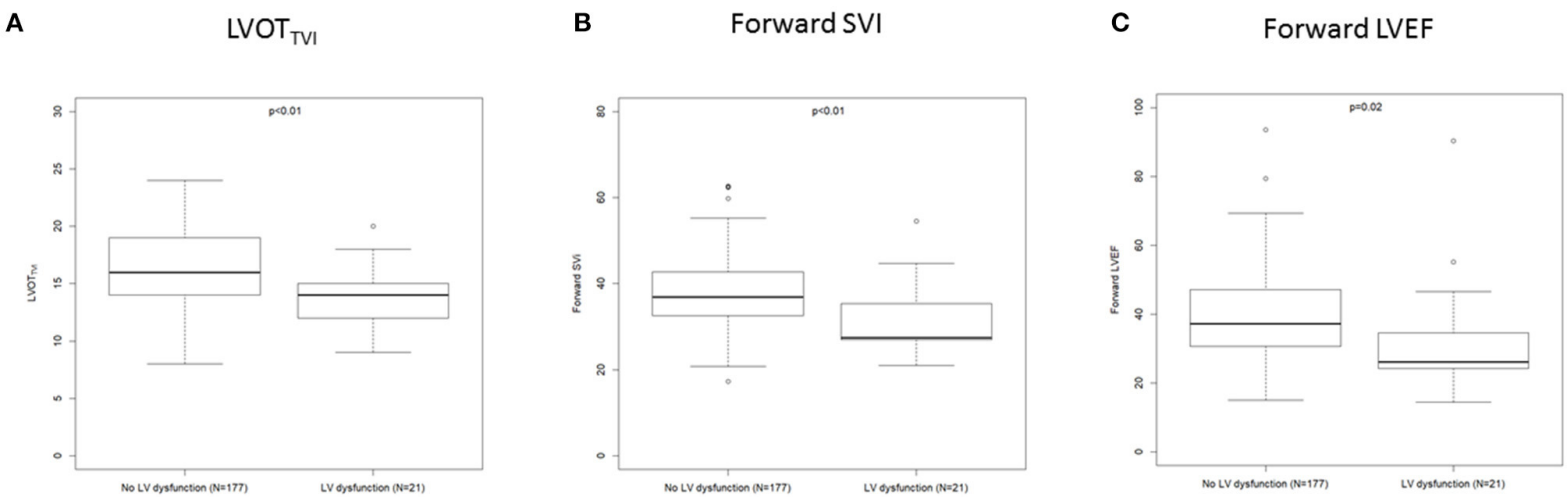
Box plot showing LVOT_TVI_ **(A)**, forward SVi **(B)**, and forward LVEF **(C)** according to the occurrence of post operative LV dysfunction.

Univariate risk factors for post-operative LV systolic dysfunction are listed in Table 3 (Figure 1).

**Table 3:** Univariate risk factor analysis for predicting post-operative LV systolic dysfunction in 198 patients undergoing mitral valve repair for severe DMR.

**Preoperative parameters**	**No post op LV dysfunction (*n* = 177)**	**Post op LV dysfunction (*n* = 21)**	**OR**	**95% CI**	***p*-value**
Age (y.o)	63.4 ± 12.3	64.1 ± 15	1.0	0.97–1.04	0.82
Male gender	132 (74.5%)	15 (71.4%)	0.85	0.32–2.51	0.75
Symptoms	38 (21.5%)	6 (28.5%)	1.46	0.49–3.87	0.46
Atrial fibrillation	18 (10.2%)	4 (19.1%)	2.08	0.55–6.38	0.23
LVEF (%)	70 [64–75]	65 [56–69]	0.91	0.86–0.96	< 0.01
LVEDD (mm)	58 [53.7–62]	60 [50–66]	1.06	0.99–1.13	0.06
LVESD (mm)	35 [30–40]	40 [35–44]	1.12	1.05–1.21	< 0.01
Indexed LVEDD (mm/m^2^)	29 [18–31]	31 [27–34]	1.14	1.02–1.27	0.02
Indexed LVESD (mm/m^2^)	19 [16–21]	23 [21–24]	1.30	1.12–1.46	< 0.02
LVEDV (mL)	187 [155–219]	197 [159–246]	1.00	0.99–1.01	0.23
LVESV (mL)	53 [41–71]	73 [52–96]	1.03	1.01–1.05	< 0.01
Indexed LVEDV (mL/m^2^)	102 [86–114]	111 [96–130]	1.01	0.99–1.03	0.13
Indexed LVESV (mL/m^2^)	29 [23–37]	41 [31–52]	1.06	1.02–1.09	< 0.01
ERO (mm^2^)	60 [48.5–80]	74 [56–94]	1.01	1.00–1.02	0.33
Regurgitant volume (mL)	90 [70–110]	92 [72–112]	1.00	0.99–1.01	0.79
LVOT_TVI_ (cm)	16 [14–19]	14 [12–15]	0.72	0.60–0.85	< 0.01
Forward SVi (mL/m^2^)	37 [32.5–42.7]	27.5 [26.9–35.4]	0.90	0.84–0.96	< 0.01
Forward LVEF (%)	37.3 [30.6–47.2]	26.1 [24.1–34.6]	< 0.01	< 0.01–0.3	0.02
LAVI (ml/m^2^)	69.5 [53.7–85]	94 [70–106]	1.03	1.01–1.05	< 0.01
sPAP	35 [28–45]	46 [30–55]	1.02	1.00–1.05	0.05

Non-normally distributed data are reported as median and interquartile ranges.

Multivariate analysis (OR [95% CI], p) revealed the following results:

▪ After adjustment for age, sex, NYHA class, LVEF, LVESD and AF, LVOT_TVI_ (0.75 [0.62–0.91], *p* < 0.01) and mean forward SVi (0.93 [0.87–0.99], *p* = 0.02) were independently associated with post-operative LV systolic dysfunction, whereas forward LVEF exhibited borderline significance (0.96 [0.92–1.00], *p* = 0.07). The impact of all classic predictors confirmed in univariate analysis disappeared when simultaneously combined with either LVOT_TVI_ or forward SVi with the exception of LVESD, which remained significant. However, the OR was lower than that of LVOT_TVI_ (Table 4).
▪ After further adjustment for LAVI, LVOT_TVI_ (0.77 [0.64–0.94], *p* = 0.008) and forward SVi (0.93 [0.87–0.99], *p* = 0.03) were independently associated with post-operative LV systolic dysfunction, whereas forward LVEF was not (0.96 [0.90–1.01], *p* = 0.27). The impact of all classic predictors confirmed in univariate analysis disappeared when simultaneously combined with either LVOT_TVI_ or forward SVi with the exception of LVESD and LAVI, which remained significant. However, the OR were lower than that of LVOT_TVI_ (Table 5).
▪ Adjustment to LV end-diastolic volume (LVESV) instead of LVESD did not change the independent impact of LVOT_TVI_ (0.77 [0.64–0.93], *p* = 0.007) and forward SVi (0.92 [0.86–0.99], *p* = 0.02), whereas forward LVEF remained nonsignificantly associated with post-operative LV dysfunction (0.96 [0.90–1.01], *p* = 0.11).

**Table 4:** Multivariable risk factor analysis for predicting post-operative LV systolic dysfunction in 198 patients undergoing mitral valve repair for severe DMR.

**Pre-operative parameters**	**OR**	**95% CI**	***p*-value**
**LVOT**_TVI_			
**LVOT**_**TVI**_	**0.75**	**0.62**–**0.91**	**< 0.01**
Age	1.02	0.98–1.06	0.42
Male	0.48	0.15–1.57	0.22
NYHA Class 3-4	1.40	0.44–4.45	0.57
LVEF	0.95	0.89–1.02	0.15
Atrial Fibrillation	2.06	0.45–9.50	0.35
LVESD	1.11	1.02–1.21	0.02
**Forward SVi**			
**Forward SVi**	**0.93**	**0.87**–**0.99**	**0.02**
Age	1.01	0.97–1.05	0.58
Male	0.56	0.18–1.77	0.32
NYHA Class 3–4	1.35	0.43–4.22	0.60
LVEF	0.95	0.89–1.01	0.11
Atrial Fibrillation	1.37	0.32–5.80	0.66
LVESD	1.10	1.01–1.20	0.02
**Forward LVEF**			
**Forward LVEF**	**0.96**	**0.92**–**1.00**	**0.07**
Age	1.01	0.97–1.06	0.49
Male	0.52	0.16–1.64	0.26
NYHA Class 3-4	1.46	0.47–4.47	0.51
LVEF	0.94	0.88–1.00	0.05
Atrial Fibrillation	1.03	0.26–4.09	0.96
LVESD	1.09	1.00–1.19	0.06

**Table 5:** Multivariable risk factor analysis for predicting post-operative LV systolic dysfunction in 198 patients subject to mitral valve repair for severe DMR after further adjustment to LAVI.

	**OR**	**95% CI**	***p*-value**
**LVOT**_TVI_			
**LVOT**_**TVI**_	**0.77**	**0.64**–**0.94**	**0.008**
Age	1.01	0.97–1.06	0.56
Male	0.49	0.14–1.67	0.25
NYHA Class 3-4	1.50	0.44–5.07	0.51
LVEF	0.95	0.89–1.02	0.15
Atrial Fibrillation	2.56	0.52–12.70	0.24
LVESD	1.10	1.01–1.21	0.03
LAVI	1.03	1.00–1.05	0.01
**Forward SVi**			
**Forward SVi**	**0.93**	**0.87**–**0.99**	**0.03**
Age	1.01	0.96–1.05	0.74
Male	0.55	0.17–1.84	0.33
NYHA class 3–4	1.44	0.43–4.82	0.54
LVEF	0.95	0.88–1.02	0.12
Atrial fibrillation	1.88	0.95–8.72	0.42
LVESD	1.09	1.00–1.20	0.05
LAVI	1.03	1.01–1.05	0.008
**Forward LVEF**			
**Forward LVEF**	**0.96**	**0.90-1.01**	**0.27**
Age	1.01	0.97–1.05	0.68
Male	0.50	0.15–1.62	0.24
NYHA class 3–4	1.56	0.48–5.06	0.45
LVEF	0.94	0.88–1.01	0.08
Atrial fibrillation	1.34	0.31–5.73	0.69
LVESD	1.09	1.00–1.20	0.05
LAVI	1.03	1.01–1.05	0.01

### Optimal thresholds for LVOT_TVI_, forward Svi, and forward LVEF

**Figure 2:**
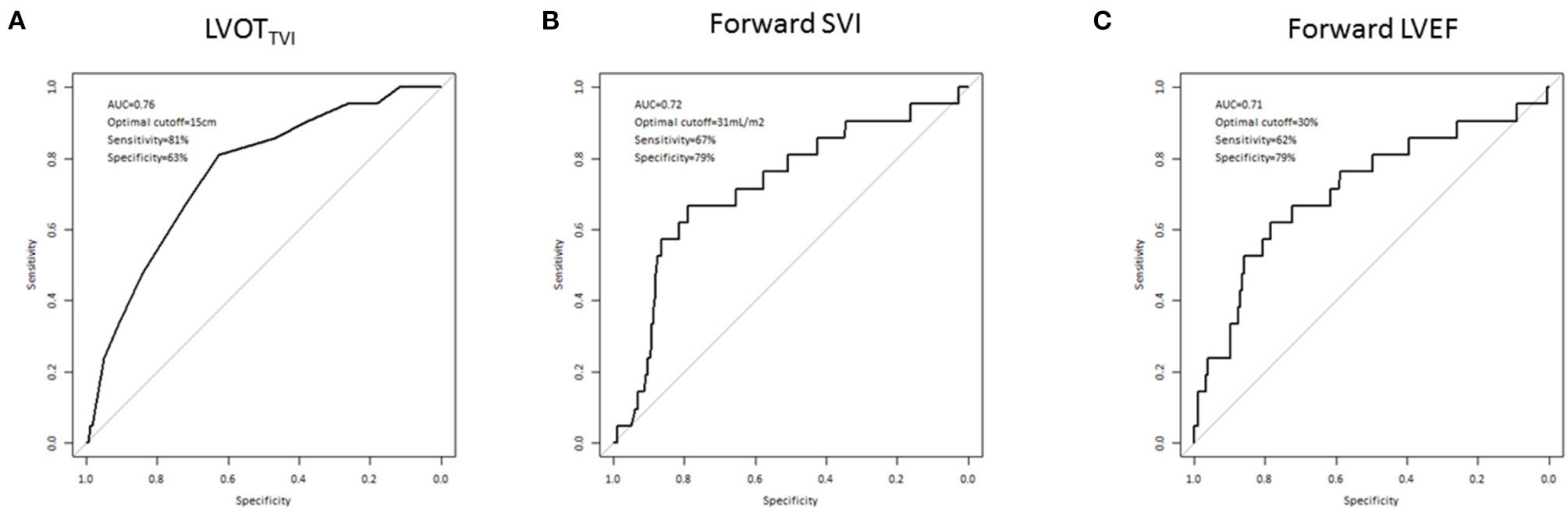
Optimal thresholds for LVOT_TVI_ **(A)**, forward SVi **(B)**, and forward LVEF **(C)** in predicting post-operative LV systolic dysfunction in 198 patients undergoing mitral valve repair for severe DMR according to ROC curve analysis.

ROC curves identified thresholds of 15 cm for LVOT_TVI_ (Se = 81% and Sp = 63%), 31 ml/m^2^ for forward SVI (Se = 67% and Sp = 79%) and 30% for forward LVEF (Se = 62% and Sp = 79%) as the most accurate in predicting post-operative LV dysfunction (Figure 2).

After adjustment for age, sex, NYHA class, LVEF, LVESD, AF, and LAVI, LVOTTVI ≤15 cm (5.97 [1.74–20.50], *p* < 0.01), forward SVI ≤31 mL/m^2^ (4.15 [1.43–12.05], *p* < 0.01) and forward LVEF ≤30% (4.02 [1.31–12.36], *p* = 0.01) were strongly and independently associated with post-operative LV systolic dysfunction. The impact of LVESD and LAVI was weaker in all models, as attested by the lower OR (Table 6, Supplementary Tables 2S, 3S, 4S).

**Table 6:** Multivariable risk factor analysis for predicting post-operative LV systolic dysfunction in 198 patients subject to mitral valve repair for severe DMR using cutoff values for LVOT_TVI_, SVi and forward LVEF defined by ROC curve analysis.

	**OR**	**95% CI**	***p*-value**
**LVOT**_TVI_			
**LVOT**_**TVI**_ **≤15**	**5.97**	**1.74–20.50**	**< 0.01**
Age	1.02	0.97–1.06	0.45
Male	0.54	0.16–1.85	0.32
NYHA class 3–4	1.63	0.48–5.49	0.43
LVEF	0.95	0.89–1.02	0.14
Atrial fibrillation	2	0.43–10	0.36
LVESD	1.12	1.02–1.22	0.01
LAVI	1.03	1.01–1.05	0.004
**Forward SVi**			
**Forward SVi** **≤31 ml/m**^2^	**4.15**	**1.43-12.05**	**< 0.01**
Age	1.01	0.96–1.05	0.77
Male	0.53	0.15–1.82	0.31
NYHA class 3–4	1.68	0.51–5.62	0.45
LVEF	0.95	0.89–1.02	0.18
Atrial fibrillation	1.81	0.38–8.33	0.45
LVESD	1.10	1.00–1.20	0.05
LAVI	1.03	1.01–1.05	0.008
**Forward LVEF**			
**Forward LVEF** **≤30%**	**4.02**	**1.31**–**12.36**	**0.01**
Age	1.01	0.97–1.05	0.72
Male	0.50	0.15–1.68	0.26
NYHA class 3–4	1.64	0.49–5.47	0.42
LVEF	0.93	0.87–1.00	0.06
Atrial fibrillation	1.26	0.28–5.55	0.75
LVESD	1.07	0.98–1.17	0.15
LAVI	1.03	1.01–1.05	0.01

### Incremental value of forward LV parameters

Among 95 asymptomatic patients with preoperative LVEF >60% and LVESD <40 mm, 5 patients (5%) developed “unexpected” LV systolic dysfunction. Among these five patients, the mean LVOT_TVI_ was 13.8 ± 2.3 cm, and four patients (80%) had an LVOT_TVI_ ≤15 cm. The forward SVi was 32.8 ± 4.5 ml/m^2^. The two patients (40%) had a forward SVi ≤31 ml/m^2^. The mean forward LVEF was 30 ± 3.1% and four patients (80%) had a forward LVEF ≤30%. All of these five patients had at least one of these markers.

## Discussion

Our study including 198 patients with MV repair for severe DMR due to MVP showed that (1) post-operative LV systolic dysfunction occurred in 11% of all patients and in 5% of asymptomatic patients despite normal preoperative classic echocardiographic LV parameters; (2) LVOT_TVI_ and forward SVI were identified as independent risk factors for post-operative LV dysfunction and appeared to be stronger predictors than classic clinical and echocardiographic markers, particularly LVEF; and (3) thresholds of 15 cm for LVOT_TVI_ and 31 ml/m^2^ for forward SVI could be early indicators of latent LV dysfunction, helping risk stratification and decision-making.

In the absence of randomized trials, DMR due to MVP has nourished a passionate controversy around the optimal timing of surgical indication in asymptomatic patients without signs of overt LV dysfunction for more than two decades. In this debate, supporters of early strategies (13, 23, 28–31) are facing guardians of the historical conservative approach (6, 32). The so-called watchful waiting approach argues that close follow-up until the occurrence of overt symptoms or patent LV dysfunction is not associated with outcome penalty based on two observational studies including small populations with likely moderate MR attested by low LV volumes (6, 32). In contrast, early surgical correction of DMR is supported by profuse observational studies, which reported a strong association between preoperative severe symptoms, decreased LVEF (16), increased LVESD (27), occurrence of AF (33) or pulmonary hypertension, and adverse outcomes both under conservative management and post-operatively (8). In addition, early repair has been associated with LA and LV function preservation (7, 8, 12, 18, 34), better post-operative outcomes than the conservative approach after propensity score matching (13, 29, 30), and restoration of normal life expectancy (35).

LV systolic dysfunction is the ultimate adverse consequence of primary MR and the first cause of post-operative mortality (5, 34). As such, its prevention remains the primary target of DMR treatment. In the compensated stage of chronic MR, LV remodeling is indeed the compensatory response to volume overload, which promotes extracellular matrix disturbances, including dissolution of collagen fibers and rearrangement of myocardial fibers. The transition to the decompensated stage of chronic MR is promoted by a reduction of the sarco-endoplasmic reticulum Ca^2+^ATP*ase2*, which is also referred to as SERCA 2, and an increase in the secretion of matrix metalloproteinases that initiates matrix proteolytic activity, cell apoptosis and ultimately impaired myocardial contractility (4). Preservation of LV systolic properties by eliminating volume overload “*on time”* therefore appears to represent the challenge in DMR management. However, assessment of LV systolic function by traditional echocardiographic indices is obscured by the modified loading conditions induced by MR. Classic markers, such as LVEF and LVESD, are pertinent indicators of patent LV dysfunction when altered (9, 10). However, these markers suffer from low sensitivity in the detection of early LV systolic impairment, which might already be present despite normal preoperative values and is subsequently unmasked post-operatively. This phenomenon is referred to as the so-called unexpected post-operative LV dysfunction (27).

Consequently, efforts have been focused on defining new surgical signals with the common objective of preserving LV function, but none have been fully validated to date. Myocardial deformation has been investigated (36), and patients with post-operative LV dysfunction have been shown to have alterations in preoperative global longitudinal strain (GLS). Although GLS seems to be more sensitive than LVEF to detect patients with LV dysfunction in severe MR, it is limited by the need for high-quality images, imperfect sensitivity and variability of GLS thresholds from one echo manufacturer to another. The detection of diffuse myocardial fibrosis by T1 mapping in MRI is currently the subject of active ongoing research and seems promising (37, 38). However, MRI suffers from its lower accessibility than TTE in routine practice. Upstream of LV parameters and mitral valve, LAVI was reported as an independent risk factor for events in patients under conservative treatment (39). However, preoperative LA remodeling is physiologically connected to the occurrence of AF and its dismal consequences both under conservative management and post-operatively (31, 40, 41). In the absence of new effective markers, current guidelines still rely on classic parameters but acknowledge their limitations and open the door for early strategies in asymptomatic patients without Class I signals as reasonable Class IIa indications (14, 15). It is however conceivable that within this subset of asymptomatic patients with normal LVEF and without extreme cavity remodeling, some could benefit from watchful waiting, whereas others should promptly undergo surgery. In this context and in an attempt to refine risk stratifications, we investigated the impact of forward flow indices in predicting the risk of post-operative LV dysfunction in DMR. Indeed, in the presence of MR and despite volume overload, global LV afterload does not increase at variance with aortic regurgitation (AR) due to the double outlet (42), and LV ejection is distributed between forward and backward flow. Forward flow in the setting of MR partly reflects the ability of myocardium to eject forward against arterial afterload instead of regurgitating backward in the low-resistance LA. As such, one can assume that markers of forward flow better reflect LV intrinsic systolic performance than LVEF and LVESD, which implicitly integrate both antegrade and retrograde streams and may overestimate LV systolic function in case of severe MR. In our study population of patients who all benefited from surgical mitral repair, the traditional impact of LVEF and LVESD in predicting post-operative LV dysfunction were actually supplanted by two forward flow indices which, decreased values could be pertinent indicators of systolic dysfunction at an early stage. LVOT_TVI_ and forward SVi were thus strong determinants of post-operative LV systolic dysfunction independent of all known risk factors for outcome. Importantly, all cases of unexpected LV dysfunction could have been detected by alteration of either one of these factors. These data are congruent with a previously published index of LV performance using LVOT_TVI_ in combination with LVESD, which suffered from mixing forward and backward markers (20). Forward LVEF did not reach significance when considered as a continuous variable but did so when the threshold of 30% was considered, which is more restrictive than the previously reported value of 50% (26). Forward LVEF has been identified as predictive of a composite criterion combining MV surgery and post-operative LV systolic dysfunction among patients with severe primary MR and normal ejection fraction (26). This parameter appeared less robust in our data for unclear reasons but might be related to a lack of power or technical issues. LVOT_TVI_ is indeed a surrogate for stroke volume (SV) that is easy to obtain in routine practice with low skill requirements in contrast to forward SVI and forward LVEF. Both of these parameters indeed require the tricky measurement of LV outflow tract diameter, the squared value of which magnifies any inaccuracy in its recording. In addition, forward LVEF requires LV tracing and perfect apical views. Despite reflecting the same physiologic concept, the increased robustness of LVOT_TVI_ observed in our study population could be the consequence of such methodological matters.

### Limitations

Given the retrospective design and relatively small study population employed in this study, these findings require validation in future large-scale prospective studies. Hypertension was present in 23% of patients, and high blood pressure values at the time of echo could have artificially decreased forward flow indices independent of LV function. However, the consistency of their predictive value in all multivariable models and their incremental values over classic signals, particularly in detecting unexpected LV dysfunction, does not favor this hypothesis. In addition, the predictive value of LVOT_TVI_ reported in this study in DMR is consistent with its known impact in the risk stratification of several cardiac diseases, including congestive heart failure (43), stable coronary artery disease and acute myocardial infarction. Decreased LVEF 6 months after mitral surgery is a debatable surrogate for irrevocable post-operative LV dysfunction, which might recover later on but was previously used as an acceptable substitution criterion (27). LVEF follow-up beyond 6 months could help refine the long-term impact of preoperative forward flow index alterations in the quest for ideal surgical signals, i.e., those that indicate surgery without compromising post-operative outcome. Such a signal does not exist in the setting of severe primary MR at present.

## Conclusion

In patients with severe DMR, forward flow parameters could represent pertinent indicators of intrinsic LV performance, and alterations in these parameters could serve as earlier markers of latent LV dysfunction compared with conventional echocardiographic indices. Among these parameters, LVOT_TVI_ appears to be the most robust and could be used as an easy recording tool in routine practice for risk stratification, thereby refining surgical indications in patients with no Class I or IIa indications.

## Data availability statement

The original contributions presented in the study are included in the article/Supplementary material, further inquiries can be directed to the corresponding author.

## Ethics statement

The studies involving human participants were reviewed and approved by APHM Assistance publique des Hopitaux de Marseille, RGPD: 2019-48 (please see Supplementary files). The patients/participants provided their written informed consent to participate in this study.

## Author contributions

All authors listed have made a substantial, direct, and intellectual contribution to the work and approved it for publication.
