# Supplementary material for: Prognostic value of forward flow indices in primary mitral regurgitation due to mitral valve prolapse
Source: Front Cardiovasc Med. 2023 Feb 23;10:1076708. doi: 10.3389/fcvm.2023.1076708 (PMC9995829; doi:10.3389/fcvm.2023.1076708)
Supplement: Supplementary file 1 [file Data_Sheet_1.pdf]

Supplementary data

Table 1S

Calibration and discrimination for each multivariable model

	Calibration	Discrimination		
	Hosmer-Lemeshow Test de Goodness of fit	AUC	IC_inf	IC_sup
Multivariable risk factor analysis for predicting postoperative LV systolic dysfunction in 198 patients undergoing mitral valve repair for severe DMR				
**$LVOT_{TVI}$**	p=0.2287	0.82	0.74	0.91
**Forward SVi**	p=0.9598	0.80	0.71	0.88
**Forward LVEF**	p=0.3532	0.80	0.71	0.90
Multivariable risk factor analysis for predicting postoperative LV systolic dysfunction in 198 patients subject to mitral valve repair for severe DMR after further adjustment to LAVI				
**$LVOT_{TVI}$**	p=0.5916	0.85	0.76	0.94
**Forward SVi**	p=0.8727	0.82	0.71	0.92
**Forward LVEF**	p=0.4176	0.81	0.71	0.91
Multivariable risk factor analysis for predicting postoperative LV systolic dysfunction in 198 patients subject to mitral valve repair for severe DMR using cutoff values for LVOTTVI, SVi and forward LVEF defined by ROC curve analysis				
**$LVOT_{TVI}$ ≤ 15**	p=0.5381	0.85	0.76	0.94
**Forward SVi ≤ 31 ml/m²**	p=0.5249	0.82	0.72	0.92
**Forward LVEF ≤ 30%**	p=0.4176	0.82	0.72	0.92

Table 2S

Baseline characteristics according to $LVOT_{TVI}$

Preoperative data	$LVOT_{TVI}$ > 15cm n = 115	$LVOT_{TVI}$ ≤ 15cm n = 83	p Value
Age (y.o)	64±12	62±13.5	0.19
Male gender	85 (73.9%)	62 (74.7%)	0.9
Symptoms	24 (20.8%)	20 (24.1%)	0.6
Atrial fibrillation	8 (6.9%)	14 (16.9%)	0.03
LVEF (%)	69.9±8.4	67.0±8.50	0.02
LVEDD (mm)	57.8±7.3	58.7±6.6	0.40
LVESD (mm)	34.5±7.5	36.4±7.2	0.08
Indexed LVEDD (mm/m^2)	32.2±4	32.2±4.2	0.09
Indexed LVESD (mm/m^2)	18.7±3.9	20.0±4.0	0.02
LVEDV (mL)	186.9±46.8	194.6±60.5	0.31
LVESV (mL)	56.4±22.4	63.2±24.9	0.04
Indexed LVEDV (mL/m^2)	100.4±21.8	106.3±29.3	0.11
Indexed LVESV (mL/m^2)	30±10.8	34.6±12.9	<0.001
ERO (mm^2)	66.3±27.1	75.6±34.4	0.11
Regurgitant volume (mL)	96±41.1	99±40	0.65
$LVOT_{VTI}$ (cm)	18.4±2.1	13.2±1.6	<0.001
Forward SVI (mL/m^2)	41.6±7.1	30.3±5.4	<0.001
Forward LVEF (%)	43.1±12.1	31.1±11.3	<0.001
Indexed LA volume (mL/m^2)	67.8±21.7	77.2±25.8	0.007
sPAP (mmHg)	38.4±13.8	41.5±17.4	0.17

Normally distributed data are reported as mean and standard deviation

Table 3S

Baseline characteristics according to Forward SVi

Preoperative data	Forward SVi > 31mL/m² n = 148	Forward SVi ≤ 31mL/m² n = 50	p Value
Age (y.o)	63.2±12.3	64.2±13.6	0.64
Male gender	109 (73.6%)	38 (76%)	0.75
Symptoms	31 (21%)	13 (26%)	0.45
Atrial fibrillation	12 (8.11%)	10 (20%)	0.02
LVEF (%)	69.8±8.3	65.5±8.4	0.03
LVEDD (mm)	57.8±6.9	59.1±7.4	0.27
LVESD (mm)	34.3±7.3	38.2±7	0.02
Indexed LVEDD(mm/m^2)	31.4±4	32.4±3.4	0.16
Indexed LVESD(mm/m^2)	18.7±3.9	20.9±3.8	0.001
LVEDV (mL)	189.8±49.6	191.1±62.6	0.87
LVESV (mL)	57.3±22.6	65.0±57.7	0.04
Indexed LVEDV (mL/ m^2)	102.7±23.7	103.2±29.8	0.89
Indexed LVESV (mL/ m^2)	30.8±11.3	35.4±13.2	0.02
ERO (mm²)	67.5±28.9	78.2±34.3	0.12
Regurgitant volume (mL)	97.2±42.8	97.3±33.5	0.98
$LVOT_{VTI}$ (cm)	17.4±2.6	12.8±1.87	<0.001
Forward SVI (mL/ m^2)	40.3±6.7	26.5±2.9	<0.001
Forward LVEF (%)	41.3±12.1	28.5±11.5	<0.001
Indexed LA volume (mL/ m^2)	70.1±22.6	76.4±27.1	0.11
sPAP (mmHg)	38.9±15.3	42.1±15.9	0.22

Normally distributed data are reported as mean and standard deviation

Table 4S

Baseline characteristics according to Forward LVEF

Preoperative data	Forward LVEF > 30% n = 145	Forward LVEF ≤ 30% n = 53	p Value
Age (y.o)	64.2±12.3	61.55±13.3	0.18
Male gender	105 (72.4 %)	42 (79.2%)	0.33
Symptoms (%)	33 (22.7%)	11(20.7%)	0.76
Atrial fibrillation (%)	16 (11%)	6 (11%)	0.95
LVEF (%)	69.2±8.8	67.4±7.5	0.18
LVEDD (mm)	56.9±6.7	61.6±6.7	<0.001
LVESD (mm)	33.8±7.1	39.7±6.6	<0.001
Indexed LVEDD (mm/m²)	31.2±4.2	33±3.6	<0.001
Indexed LVESD(mm/m²)	18.5±3.9	21.2±6.7	<0.001
LVEDV (mL)	172.7±41.4	237.8±52.4	<0.001
LVESV (mL)	52.6±19.6	77.3±24.4	<0.001
Indexed LVEDV (mL/m²)	94.1±20.3	126.7±22.06	<0.001
Indexed LVESV (mL/m²)	28.5±9.8	41.3±12.4	<0.001
ERO (mm²)	63.7±24.5	89.7±38.4	<0.001
Regurgitant Volume (mL)	89.3±36.2	119.8±45	<0.001
$LVOT_{VTI}$ (cm)	17.1±2.7	13.7±2.9	<0.001
Forward SVI (mL/m²)	39.5±7.8	29.7±5.6	<0.001
Forward LVEF (%)	43.3±11.3	23.7±4.1	<0.001
Indexed LA volume (mL/m²)	69.1±23.5	79.1±23.8	0.01
sPAP (mmHg)	37.4±14.3	45.6±16.7	0.001

Normally distributed data are reported as mean and standard deviation
